# Online ion-exchange chromatography for small-angle X-ray scattering

**DOI:** 10.1107/S2059798316012833

**Published:** 2016-09-15

**Authors:** Stephanie Hutin, Martha Brennich, Benoit Maillot, Adam Round

**Affiliations:** a Institut de Biologie Structurale, 71 Avenue des Martyrs, 38042 Grenoble, France; b European Synchrotron Radiation Facilty, 71 Avenue des Martyrs, 38042 Grenoble, France; c Integrated Structural Biology Department, Institut de Génétique et de Biologie Moléculaire et Cellulaire, BP 10142, 67404 Illkirch, France; d EMBL Grenoble Outstation, 71 Avenue des Martyrs, 38042 Grenoble, France; e EPSAM, Keele University, Keele, England

**Keywords:** BioSAXS, ion-exchange chromatography, online purification

## Abstract

Biological small-angle X-ray scattering (BioSAXS) is a powerful technique to determine the solution structure, particle size, shape and surface-to-volume ratio of macromolecules. However, a drawback is that the sample needs to be monodisperse. To ensure this, size-exclusion chromatography (SEC) has been implemented on many BioSAXS beamlines. Here, the integration of ion-exchange chromatography (IEC) using both continuous linear and step gradients on a beamline is described. Background subtraction for continuous gradients by shifting a reference measurement and two different approaches for step gradients, which are based on interpolating between two background measurements, are discussed. The results presented here serve as a proof of principle for online IEC and subsequent data treatment.

## Abstract 2

SAXS coupled with online ion-exchange chromatography allows the collection of high-quality BioSAXS data.

## 1. Introduction   

Biological small-angle X-ray scattering (BioSAXS) can reveal solution structures in terms of the average particle size and shape of biological macromolecules, as well as information on the surface-to-volume ratio (Graewert & Svergun, 2013 ▸; Kikhney & Svergun, 2015 ▸; Putnam *et al.*, 2007 ▸; Jacques & Trewhella, 2010 ▸). This method is an accurate, mostly non­destructive approach, which requires little sample preparation compared with that required for other structural biology techniques, such as crystallography. However, for accurate interpretation the sample is required to be monodisperse; this requirement is often a problem as biological macromolecules can be susceptible to aggregation. Recently, the combination of online size-exclusion chromatography (SEC) and SAXS has been implemented directly on beamlines in order to overcome this obstacle, to ensure data quality and to make this technique more accessible for increasingly difficult samples (Lambright *et al.*, 2013 ▸; Round *et al.*, 2013 ▸; David & Pérez, 2009 ▸; Graewert *et al.*, 2015 ▸; Mathew *et al.*, 2004 ▸; Watanabe & Inoko, 2009 ▸; Acerbo *et al.*, 2015 ▸; Grant *et al.*, 2011 ▸). Additional techniques, such as time-resolved (TR) SAXS experiments using desalting columns for quick buffer exchanges (Jensen *et al.*, 2010 ▸) and differential ultracentrifugation, have been successfully coupled with SAXS (Hynson *et al.*, 2015 ▸). However, even when using SEC–SAXS, samples can still be difficult to measure, for example when different species do not separate. This can be owing to their size (the difference in molecular mass should be at least 10%), owing to the limited resolution range of the SEC column or owing to the physical properties of the sample such as hydrophobic surfaces, flexibility or lack of stability, *e.g.* separation of complexes into individual protomers. In these cases, data collection, analysis and interpretation can be difficult.

Another common problem is the quantity of sample needed. Many proteins are difficult to express or purify and the required quantity (about 100 µl at 3 mg ml^−1^) of monodisperse sample cannot be obtained. For SEC–SAXS measurements, the high degree of dilution on the column [up to a factor of ten depending on the column type and sample (Watanabe & Inoko, 2009 ▸; Kirby *et al.*, 2013 ▸)] requires even more concentrated samples (5 mg ml^−1^ or above), although depending on the column less volume (down to 10 µl) might be sufficient. Even if the samples are soluble at these concentrations, they are not always stable and can often aggregate.

An alternative purification method is ion-exchange chromatography (IEC). IEC separates ionized molecules based on their total net surface charge, which changes gradually with the pH and/or the salt concentration of the buffer (Karlsson & Hirsh, 2011 ▸). This technique enables the separation of molecules of similar size, which can be difficult to separate by other techniques. In general, if the net surface charge of a protein is higher (positive or negative) than that of the IEC resin (anion or cation exchange, respectively), the protein will bind to it. When using a linear salt gradient to increase the charge in the mobile phase, a specific protein will be eluted at a specific salt concentration. Given that different oligomeric states and aggregates differ in their surface area, and hence their surface-charge distribution, it is also possible to separate oligomeric species (Kluters *et al.*, 2015 ▸).

IEC has the advantage of providing moderate resolution and, when working with large volumes of dilute samples, concentrating the sample, since the eluate concentration is determined by the capacity of the column and the binding affinity of protein to the column, and not by the initial concentration. Therefore, it is possible to store and transport samples at low concentrations prior to the IEC experiment. Additionally, many IEC columns perform well at flow rates in the millilitre per minute range, which limits the risk of collecting data from radiation-damaged material. However, IEC does require some optimization regarding the pH and the salt gradient (Yigzaw *et al.*, 2009 ▸), which can and should be performed prior to any SAXS-coupled data collection.

Here, we present a proof of principle for an alternative to SEC–SAXS measurements using ion-exchange chromatography (Selkirk, 2004 ▸) online with the SAXS experiment. This has been implemented and tested on the BioSAXS beamline BM29 at the European Synchrotron Radiation Facility (ESRF) in Grenoble, France (Pernot *et al.*, 2013 ▸).

## 2. Materials and methods   

### 2.1. Purification of BSA on an ion-exchange column   

For the preparative offline IEC experiment, 100 µl of a 77 mg ml^−1^ BSA (lyophilized powder, essentially globulin-free; Sigma) solution in buffer *A* [20 m*M* Tris pH 7, 25 m*M* NaCl, 5% glycerol, 1 m*M* dithiothreitol (DTT)] was prepared and injected onto a Uno Q-1R (Bio-Rad) column on a Biologic system (Bio-Rad) equilibrated with buffer *A*. The protein concentration was determined by measuring the absorption at 280 nm with a NanoDrop (NanoDrop 1000, Thermo Fisher) using a mass extinction coefficient of 6.7 for a 1% (10 mg ml^−1^) BSA solution. The high concentration enables easy injection onto the column using injection loops. A salt gradient was made by mixing buffer *A* with buffer *B* (20 m*M* Tris–HCl pH 7, 1 *M* NaCl, 5% glycerol, 1 m*M* DTT). The flow rate for the offline experiment was 2 ml min^−1^.

For IEC–SAXS experiments, only 50 µl was injected using the autosampler of the HPLC system (SIL-20ACXR) and the flow rate was 1 ml min^−1^.

Peak fractions from the preparative run (20 µl per 1500 µl fraction size) were analysed on a 12% SDS–PAGE stained with InstantBlue (Expedeon). PageRuler Plus Prestained Protein Ladder (Thermo Fisher) was used as a molecular-weight marker.

### 2.2. D5_323–785_ cloning, expression and purification   

A fragment of the helicase–primase D5R representing the D5N and helicase domains, D5_323–785_, was cloned, expressed and purified as described in Hutin *et al.* (2016 ▸). The construct was cloned into the pProEx HTb vector (Life Technologies) using the primers 5′-GCGCCATGGGTAATAAACTGTTT­AATATTGCAC-3′ and 5′-ATGCAAGCTTTTACGGAGATGAAATATCCTCTATGA-3′ and expressed in *Escherichia coli* BL21 (DE3) Star cells (Novagen). The bacterial pellet was resuspended in lysis buffer (50 m*M* Tris–HCl pH 7, 150 m*M* NaCl, 5 m*M* MgCl_2_, 10 m*M* β-mercaptoethanol, 10% glycerol) with cOmplete protease-inhibitor cocktail (Roche) and 1 µl benzonase per 10 ml and lysed by sonication. The supernatant was loaded onto a nickel-affinity column (HIS-Select; Sigma), which was washed with 10 column volumes (CV) of lysis buffer, 10 CV washing buffer (50 m*M* Tris–HCl pH 7, 1 *M* NaCl, 10 m*M* β-mercaptoethanol, 10% glycerol) and 10 CV imidazole wash (50 m*M* Tris–HCl pH 7, 150 m*M* NaCl, 10 m*M* β-mercaptoethanol, 10% glycerol, 30 m*M* imidazole). D5_323–785_ was eluted in 20 m*M* Tris–HCl pH 7, 150 m*M* NaCl, 10 m*M* β-mercaptoethanol, 10% glycerol, 200 m*M* imidazole and the buffer was exchanged back to the lysis buffer on an Econo-Pac 10DG Desalting column. His-TEV cleavage was performed at room temperature overnight and the cleaved protein was passed over a second nickel column before injection onto a Superose 6 column (GE Healthcare) equilibrated with gel-filtration buffer (20 m*M* Tris–HCl pH 7, 150 m*M* NaCl, 10% glycerol, 1 m*M* DTT). The eluted peak fractions of D5_323–785_ were then combined and diluted to 25 m*M* NaCl and 5% glycerol by keeping the buffer concentration equal. 30 ml of the sample, containing about 5 mg of protein, were then loaded onto a Uno Q-1R column (Bio-Rad; buffer *A*, 20 m*M* Tris pH 7, 25 m*M* NaCl, 5% glycerol, 1 m*M* DTT; buffer *B*, 20 m*M* Tris pH 7, 1 *M* NaCl, 5% glycerol, 1 m*M* DTT). The protein concentration was estimated by measuring the absorption at 280 nm with a NanoDrop (NanoDrop 1000, Thermo Fisher) using a mass extinction coefficient of 8.38 for a 1% (10 mg ml^−1^) D5_323–785_ solution. For offline IEC the flow rate used was 2 ml min^−1^, which was reduced to 1 ml min^−1^ in the online experiments. Glycerol was added to all buffers as a co-solvent to enhance the stability of D5_323–785_ in aqueous solution in order to prevent protein aggregation.

As for BSA, fractions from the preparative run were analysed by SDS–PAGE.

### 2.3. IEC–SAXS data collection   

SAXS data were collected on BM29 at ESRF (Pernot *et al.*, 2013 ▸) using a PILATUS 1M detector (Dectris) at a distance of 2.864 m from the 1.8 mm diameter flowthrough capillary. The scattering of pure water was used to calibrate the intensity to absolute units (Orthaber *et al.*, 2000 ▸). The intensities were scaled such that the forward scattering corresponds directly to the concentration (in mg ml^−1^) times the molar mass (in kDa) of idealized proteins, *i.e.* 1 a.u. = 8.03 × 10^−4^ cm^−1^, unless explicitly stated otherwise. Data collection was performed continuously throughout the chromatography run at a frame rate of 1 Hz. The X-ray energy was 12.5 keV and the accessible *q*-range was 0.032–4.9 nm^−1^. The incoming flux at the sample position was of the order of 5 × 10^11^ photons s^−1^ in 700 × 700 µm. A summary of the acquisition parameters is given in Table 1 ▸. All images were automatically azimuthally averaged with *pyFAI* (Ashiotis *et al.*, 2015 ▸; Kieffer & Wright, 2013 ▸; Kieffer & Karkoulis, 2013 ▸).

Online purification was performed with a high-pressure liquid-chromatography (HPLC) system (Shimadzu, France) consisting of an inline degasser (DGU-20A5R), a binary pump (LC-20ADXR), a valve for buffer selection and gradients, an auto-sampler (SIL-20ACXR), a UV–Vis array spectrophotometer (SPD-M20A) and a conductimeter (CDD-10AVP). The HPLC system was directly coupled to the flowthrough capillary of the SAXS exposure unit (Round *et al.*, 2015 ▸). The flow rate for all online experiments was 1 ml min^−1^, resulting in a mean passage time of material through the X-ray beam of 0.1 s.

Initial evaluation of the data quality was performed using the automatic SEC–SAXS processing pipeline available at the BM29 beamline (Brennich *et al.*, 2016 ▸; De Maria Antolinos *et al.*, 2015 ▸).

### 2.4. Background subtraction for BSA on a linear gradient   

In order to subtract an appropriate background, the method developed by Hynson and coworkers for differential ultracentrifugation (Hynson *et al.*, 2015 ▸) was applied. Similarly to the salt gradient in IEC–SAXS used here, the background signal in differential ultracentrifugation-coupled SAXS changes owing to a sucrose gradient. The background-correction method of Hynson and coworkers identifies the appropriate background subtraction as that which provides a stable SAXS signal throughout the peak. The stability of the signal can be assessed by comparing the ratio of scattering in a low-*q* region to that in a mid-*q* region: for a stable signal this ratio is constant, whereas for an unstable signal it changes throughout the peak. Incorrect background correction results in a systematic change in the scattering signal depending on the protein concentration. The choice of the regions depends to some extent on the protein of interest and in particular on its size. However, we found that the regions used by Hynson and coworkers (0.11–0.5 and 1.5–2.5 nm^−1^ for low-*q* and mid-*q* regions, respectively) suited well: the low-*q* region reflects the correct overall size of the protein, whereas most of the characteristic features of BSA are present in the mid-*q* region (see, for example, Fig. 5*d*).

In Hynson *et al.* (2015 ▸) the offset between the individual sample and buffer runs is first determined by matching the high-*q* region of the scattering profile. This shift is then fine tuned by assessing the variability of the low-*q* to mid-*q* ratio for a fine grid of interpolated buffers.

**Figure 1:**
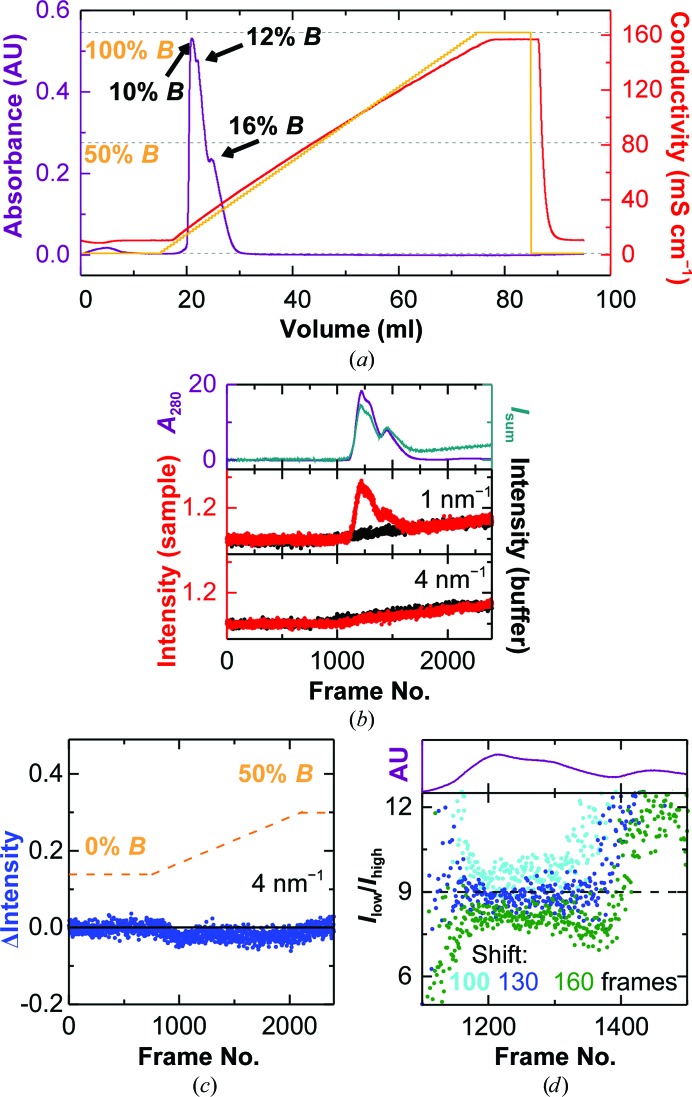
Linear gradient IEC–SAXS performed on BSA. (*a*) Standard ion-exchange chromatogram of BSA on the Uno Q-1R column, highlighting the locations of the three peaks. Orange line, percentage of buffer *B*; red line, resulting conductivity; violet line, absorbance at 280 nm. (*b*) IEC–SAXS chromatograms of BSA using a linear salt gradient. Top panel, UV absorbance (violet) and total scattering intensity (green). Middle and bottom panels, chromatograms of sample (red) and buffer (black) runs at *q* = 1 nm^−1^ (middle) and 4 nm^−1^ (bottom). (*c*) Difference between the scattering intensities measured at *q* = 4 nm^−1^ in the sample and buffer runs (blue symbols). The dotted orange line illustrates the salt gradient. (*d*) Comparison of the scattering ratio (0.11–0.5 nm^−1^ *versus* 1.5–2.5 nm^−1^) for background correction using a shift of 100 frames (cyan), 130 frames (blue) and 160 frames (green) between the sample and buffer runs. The dotted black line serves as a visual aid for a constant line.

In IEC–SAXS experiments, the overall change as well as the difference between individual frames in the background signal throughout the gradient and the offset between the buffer and sample measurements are much smaller than in the sucrose gradient used in the ultracentrifugation experiment [compare the bottom part of Fig. 1 ▸(*b*) in this paper with Fig. 2(*a*) in Hynson *et al.* (2015 ▸)]. As a consequence, the approach can be slightly simplified: the optimal shift between the recorded frames can be determined directly without the need for interpolation. To achieve this, we shift the buffer run with respect to the sample run in steps of five frames and subtract frame-wise. The low-*q* to mid-*q* ratio is then calculated for each frame and the variability in the region of interest is assessed. The perfect shift would result in a parallel line in the ratio *versus* frame-number plot throughout the region, whereas systematic under-subtraction results in a concave shape and over-subtraction in a convex shape.

Using this approach, we can determine the best shift with an error of ten frames. This might seem less accurate in comparison with the subframe precision of Hynson and coworkers, but the difference between individual buffer frames in an IEC gradient is much lower than in differential ultracentrifugation [a CORMAP test (Franke *et al.*, 2015 ▸) on 20 frames in the region of interest showed no significant deviation]. This implies that the effect of these ten frames on the subtraction is small.

### 2.5. SAXS data analysis   

To compare the scattering from different frames, the ratio of scattering in the low-*q* region (0.11–0.5 nm^−1^) to the high-*q* region (1.5–2.5 nm^−1^) was calculated in the same manner as in Hynson *et al.* (2015 ▸). For easier comparison between different buffer subtractions, the result was normalized to give the same mean over the region of interest. Radii of gyration were calculated using *AUTORG* from the *ATSAS* package (Petoukhov *et al.*, 2007 ▸), *P*(*r*) functions were calculated using *GNOM* (Svergun, 1992 ▸) and Porod volumes were estimated using the Volume interface of *SCÅTTER* (available at http://www.bioisis.net/tutorial/9). The estimation of molecular mass based on the Porod analysis was performed by dividing the Porod volume by 1.7 nm^3^ kDa^−1^ (Petoukhov *et al.*, 2007 ▸). All other manipulations were performed in the Spyder interface for Python 3.4 using the *NumPy* and *SciPy* packages (Jones *et al.*, 2001 ▸). The scripts used are available at https://github.com/maaeli/IEC. As the background scattering is dependent on the buffer composition, the gradient or stepwise buffer changes during elution must be accounted for in the subtraction process. We present three methods to find the optimal background subtractions, which are explained for the individual cases in §[Sec sec3]3.

Known BSA crystal structures were compared with the experimental data using *CRYSOL* (Svergun *et al.*, 1995 ▸). Default fitting parameters were used, which allow the hydration shell to be adjusted to optimize the fit.

## 3. Results and discussion   

### 3.1. IEC–SAXS of bovine serum albumin using a linear salt gradient   

The first test case was bovine serum albumin (BSA), using a standard linear salt gradient for elution and a blank run of the same gradient for buffer subtraction. We chose BSA as it is known to form higher oligomers and aggregates in solution (Folta-Stogniew & Williams, 1999 ▸), allowing us to assess the ability of the method to reduce sample polydispersity.

**Figure 2:**
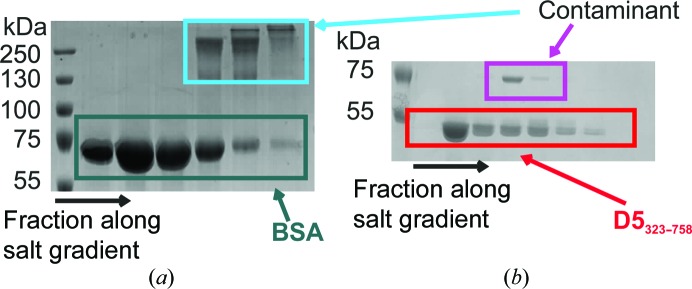
(*a*) 10% SDS–PAGE of fractions of BSA along the salt gradient collected after the Uno Q-R1 column run. Pure BSA is indicated in teal and the contaminant is indicated in cyan. The protein ladder is labelled in kDa. (*b*) Fractions from a Uno Q-R1 column run of D5_323–785_ along the salt gradient. The red box frames the D5_323–785_ protein, while the magenta box indicates the contaminant.

We performed a standard BSA run on the ion-exchange column using an offline FLPC system, evaluated the chromatogram and observed several peaks in the first third of the chromatogram (Fig. 1 ▸ *a*). Their approximate buffer *B* percentages were determined to be 10, 12 and 16%, corresponding to NaCl concentrations of 122.5, 142 and 181 m*M*, respectively. SDS–PAGE of different fractions throughout the peak confirmed that the first two peaks correspond to BSA, while the third peak is a higher molecular-mass contaminant (Fig. 2 ▸ *a*).

We then ran the same sample online on the BM29 beamline at ESRF, as described in §[Sec sec2]2, and continuously acquired SAXS data from the eluent at a rate of 1 Hz.

**Figure 3:**
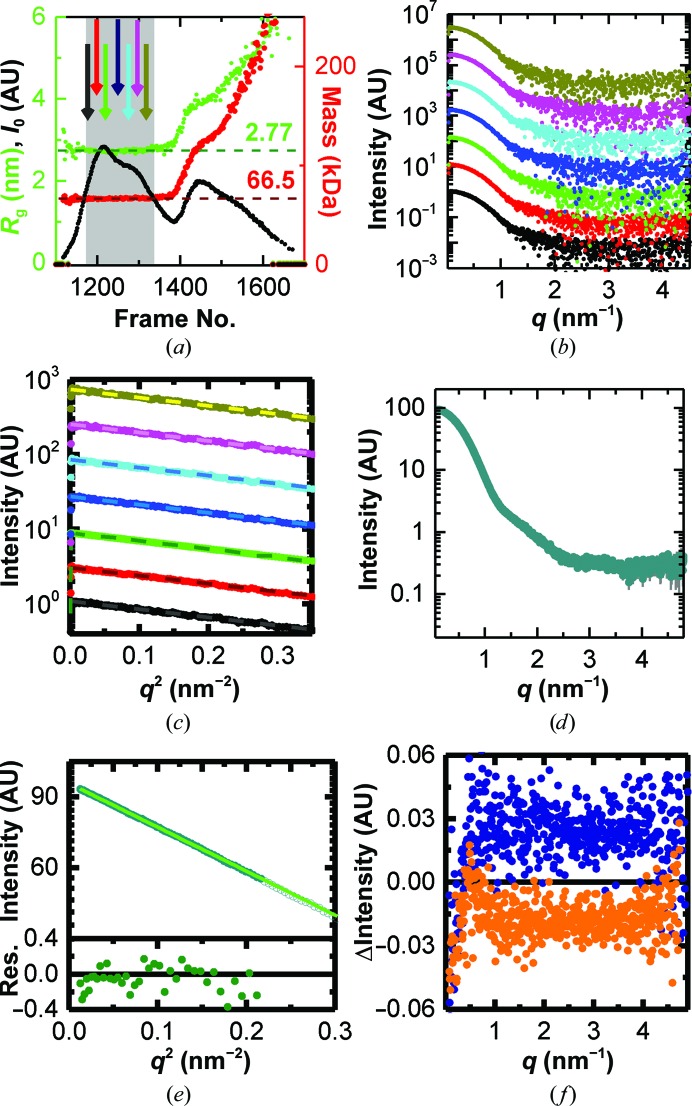
Background analysis of linear gradient IEC–SAXS on BSA. (*a*) Forward scattering (black), radius of gyration (green) and mass (red) based on the correlated volume for the background-corrected curves in the region of interest. The dotted lines correspond to the expected radius of gyration (dark green) and molecular mass (dark red) of BSA. The grey area indicates which frames were used for subsequent averaging; the arrows indicate the positions of the individual curves shown in (*b*) and (*c*). (*b*) Individual scattering curves and (*c*) Guinier plots at different positions throughout the chromatogram (the curves are offset for clarity). The colour code and positions in the chromatogram are as indicated in (*a*). (*d*) Averaged scattering curve from the region indicated in grey in (*a*). (*e*) Guinier fit (light green line) of the scattering curve shown in (*d*). Open symbols represent points that were not used for fitting. The lower panel shows the residuals. (*f*) Differences in scattering between the curve found by shifting the buffer run by 130 frames and a clearly under-subtracted curve (120 frames shift, orange) and over-subtracted curve (160 frames shift, blue).

In order to improve the resolution of the peaks and to ensure a sufficiently high sampling rate, the flow rate for the online experiments was reduced to 1 ml min^−1^. The UV absorption signal at 280 nm and the total scattering elution profiles show the expected three peaks (Fig. 1 ▸ *b*, top), whereas the scattering at higher angles increases linearly (Fig. 1 ▸ *b*, bottom). The matching background was found to be slightly shifted with respect to the buffer run (compare the bottom graph in Fig. 1 ▸ *b* with Fig. 1 ▸ *c*). It is therefore necessary to subtract not the directly corresponding frames but slightly shifted ones. Possible reasons for this shift are a nonperfect synchronization between the ion-exchange run and the SAXS data acquisition or the co-elution of ions bound to the column. To identify the optimal shift, as described in §[Sec sec2]2, we applied the approach developed by Hynson *et al.* (2015 ▸) for differential ultracentrifugation coupled to SAXS. For each shift the ratio of scattering at low *q* (0.11–0.5 nm^−1^) *versus* mid-to-high *q* (1.5–2.5 nm^−1^) is calculated for each frame. If the sample scatters in the same way throughout the peak, this value should not change and a ‘constant’ value over the peak indicates a suitable background subtraction. Fig. 1 ▸(*d*) shows how the ratio changes in the region of interest for different values of the shift. For the first two peaks the ideal shift was found to be 130 ± 10 frames, which corresponds to about 2.2 ml. In addition, the ratio was constant over both peaks, indicating that one species elutes in a double peak. However, for the third peak no shift giving a flat line was found, indicating that the scattering from the sample is not constant throughout the peak and that the peak therefore represents more than one species. The SDS–PAGE from the offline run shows that in this peak BSA is contaminated by a larger protein species (Fig. 2 ▸ *a*). The forward scattering and the radius of gyration obtained using *AUTORG* (Petoukhov *et al.*, 2007 ▸) were calculated from subtracted curves and the mass was estimated *via* the correlated volume approach (Rambo & Tainer, 2013 ▸). Fig. 3 ▸(*a*) confirms that, as expected for a single species, both the mass and the radius of gyration are constant throughout the initial double peak. Based on the forward scattering intensity (Mylonas & Svergun, 2007 ▸), and assuming monomeric BSA (66.5 kDa), the maximum protein concentration throughout the peak was estimated at 1.75 mg ml^−1^, which is well below the regime in which significant structure-factor contributions to the signal would become relevant (Skou *et al.*, 2014 ▸; Zhang *et al.*, 2007 ▸). An additional comparison of individual frames throughout the double peak shows that the SAXS signal is identical throughout the peak, with the lowest adjusted *p*-value in a CORMAP test (Franke *et al.*, 2015 ▸) being 0.16 (Figs. 3 ▸ *b* and 3 ▸ *c*). This suggests that the two subpeaks do not represent different conformations of BSA. 169 frames were averaged in the region of interest shown (grey) in Fig. 3 ▸(*a*). The resulting curve (Fig. 3 ▸ *d*) gives a radius of gyration of 2.7 ± 0.1 nm (Fig. 3 ▸ *e*) and a Porod volume of 116 ± 5 nm^3^, corresponding to a molecular weight of about 68 kDa (Petoukhov *et al.*, 2007 ▸; see Table 1 ▸ for further details). Both of these values correspond well to the expected size (2.77 nm; PDB entry 3v03; Majorek *et al.*, 2012 ▸) and molecular weight (66.5 kDa) of monomeric BSA. Additional comparison to the monomeric crystal structure (PDB entry 3v03; Majorek *et al.*, 2012 ▸) shows a similarly good match as the SEC–SAXS data for BSA (χ^2^ = 1.82; Fig. 5*d* and Supplementary Fig. S1*b*). To estimate to what extent an incorrect shift affects these results, the corresponding averages for shifts of 120 and 140 frames, respectively, were calculated. The absolute differences from the 130-frame shift are shown in Fig. 3 ▸(*f*). In both cases, above 1 nm^−1^ the curve is shifted by a small constant, which many modelling algorithms take into account (Knight & Hub, 2015 ▸; Petoukhov *et al.*, 2007 ▸; Svergun *et al.*, 1995 ▸). Below 1 nm^−1^ differences in the relative contribution of capillary scattering result in a *q*-dependent difference, which could in principle affect modelling. However, these differences contribute less than 0.2% to the scattering signal and thus their influence is negligible.

### 3.2. IEC–SAXS of bovine serum albumin using a stepwise salt gradient   

The linear gradient IEC works well for many proteins, but for samples where the elution peak is broad or is not well separated from other peaks, manual selection of appropriate steps in the gradient can ensure a purer and higher peak on faster timescales. For these cases, we describe an elution system in which the salt concentration is increased in predefined steps. The advantage of this approach is that by choosing a sufficiently long step length, it is possible to use buffer measurements from the same chromatography run for background correction, reducing the risk of nonmatching buffers owing to slow drifts. On the downside, even if the change in the buffer mixing ratio at the pumps is instantaneous, various effects, such as Taylor dispersion of flow in the capillary (see, for example, Wunderlich *et al.*, 2014 ▸), co-elution of small molecules from the columns and the creation of new interaction sites for salt on the column and the eluted protein, result in non-instantaneous gradients at the measurement position. To show the validity of this approach, we again used BSA with the salt concentrations determined above from the offline IEC run.

**Figure 4:**
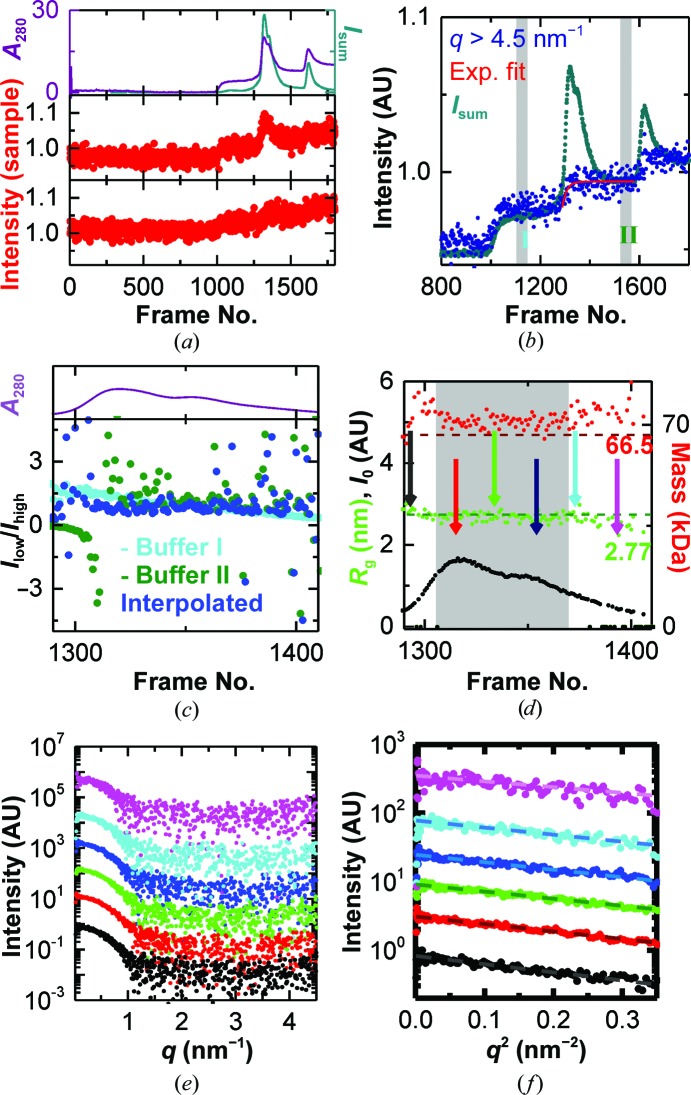
Stepwise-gradient IEC–SAXS performed on BSA. (*a*) IEC–SAXS chromatograms of BSA using a stepwise salt gradient. Top panel, UV absorbance (violet) and total scattering intensity (green). Middle and bottom panels, chromatograms at 1 and 4 nm^−1^, respectively. (*b*) The buffer signal (mean scattering above 4.5 nm^−1^, blue) increases slowly during the elution of the peak (total scattering intensity, green) and can be modelled by an exponential decay (red) between the buffer before the peak (I) and after the peak (II). (*c*) Comparison of the scattering ratio (0.11–0.5 nm^−1^ *versus* 1.5–2.5 nm^−1^) for background correction using the buffer before the peak (I, cyan), after the peak (II, green) and the modelled buffer (blue). (*d*) Forward scattering (black), radius of gyration (green) and mass (red) based on the correlated volume for the background-corrected curves in the peak region. The grey area indicates the frames that were used for subsequent averaging; the arrows indicate the positions of the individual curves presented in (*e*) and (*f*). (*e*) Individual scattering curves and (*f*) Guinier plots at different positions throughout the chromatogram (curves are offset for clarity). The colour code and the positions in the chromatogram are as indicated in (*d*).

The elution steps were chosen in such a way that the first two subpeaks of the BSA elute in the same step. In this case, BSA elutes as a single peak (Fig. 4 ▸ *a*). As expected, the background scattering at high angles does not increase instantaneously, but saturates slowly after a steep initial increase. In order to subtract an appropriate background from each measured frame, it is necessary to interpolate between the two buffers recorded before and after the peak. As the scattering of BSA at high angles is rather low and the increase in signal above *q* = 4.5 nm^−1^ does not follow the protein concentration (Fig. 4 ▸ *a*), one can assume that the changes in signal in this region are only owing to the difference in background. In order to limit the effect of the rather high noise level in this region, the increase is modelled with a continuous function instead of using the high-*q* region of each frame to interpolate the buffer individually. A least-parameter model for an asymmetric, saturating increase as observed here is a single exponential decay from the buffer before (region I) to the buffer after (region II) the peak, *i.e.* a fit to II − (II − I)exp[−(*N* − *N*_0_)/*N*′], where *N* is the frame number, *N*_0_ is a constant offset and 1/*N*′ is the rate of increase. To obtain the correct values for *N*_0_ and *N*′, the mean of the data was fitted above 4.5 nm^−1^ (Fig. 4 ▸ *b*). The best fit is obtained with *N*_0_ = 1289 ± 2 and *N*′ = 17 ± 3, giving an χ^2^ of 0.86. These parameters match the starting point of the increase (frame 1290) and the estimated half-life of 12 ± 2 frames well and allow us to model the entire *q*-range.

**Figure 5:**
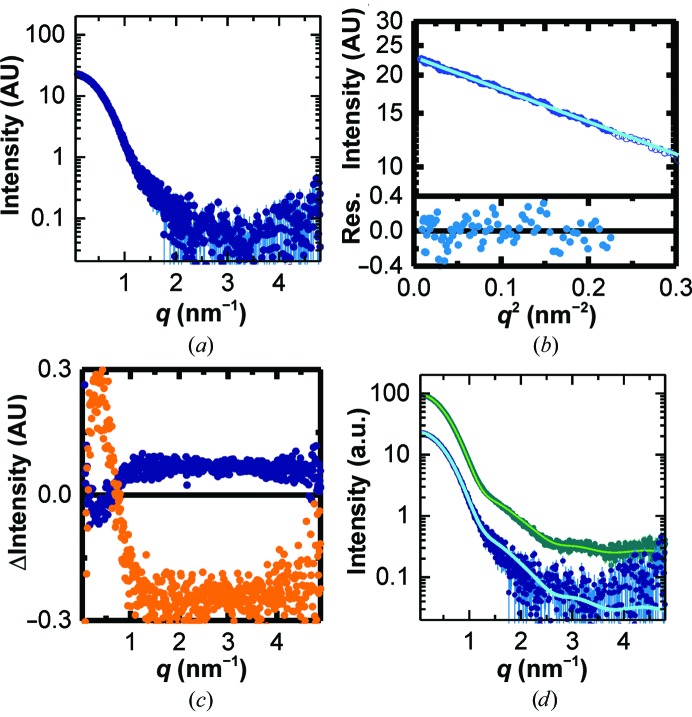
Stepwise-gradient IEC–SAXS analysis of averaged BSA data. (*a*) Scattering curve based on averaging of the region indicated in grey in Fig. 4(*d*). (*b*) Guinier fit (light blue line) of the scattering curve shown in (*a*). Open symbols represent points that were not used for fitting. The lower panel shows the residuals. (*c*) Differences in scattering between the curve found by interpolating the buffer and a clearly under-subtracted curve (average of buffers from regions I and II, orange) and over-subtracted curve (buffer from region II, blue). (*d*) Fits to the monomeric crystal structure of BSA (PDB entry 3v03; Majorek *et al.*, 2012 ▸) eluted with a linear gradient (green) and a stepwise gradient (blue).

This procedure allows the background to be subtracted individually for each frame and a constant scattering from the sample throughout the peak to be confirmed by assessing the stability of the ratio of scattering at low *q versus* mid/high *q*, as discussed above (Figs. 4 ▸ *c* and 4 ▸ *d*). Individual comparison of frames confirmed this constant signal (Figs. 4 ▸ *e* and 4 ▸ *f*). Based on the forward scattering, the maximum concentration in this case was only 0.35 mg ml^−1^. After averaging (Fig. 5 ▸ *a*), a scattering curve which corresponds to monomeric BSA (χ^2^ = 3.63; Fig. 5 ▸ *d*) was found. The radius of gyration is 2.7 ± 0.1 nm (Fig. 5 ▸ *b*) and the Porod volume is 116 ± 5 nm^3^, matching previous results (also see Table 1 ▸). This shows that important structural parameters can be determined using either linear or stepwise gradients.

In order to estimate the mis-subtraction of the buffer and its effect on the conclusions that can be drawn from the data, one needs to determine corresponding over-subtracted and under-subtracted curves. We decided to over-subtract by directly subtracting buffer II and to under-subtract by subtracting the mean of buffer I and buffer II. This choice might be more extreme than strictly necessary, but provides an upper limit of the effect of the mis-subtraction. The differences in this case (Fig. 5 ▸ *c*) are much more pronounced than for a linear gradient case (Fig. 3 ▸ *f*). This means that the background subtraction for the linear case is more accurate.

Above 1 nm^−1^, buffer mismatch causes a constant shift in the signal. Below 1 nm^−1^, the differences are larger but do not impact the structural parameters determined from this region, with the radius of gyration remaining at 2.7 ± 0.1 nm and the Porod volume increasing within its error to 120 ± 5 nm^3^ only for the subtraction of buffer II and remaining unchanged for the other case.

This implies that while interpretations of domain arrangements might be affected by buffer mismatches, the observed overall shape (size, anisotropy, …) of the protein can still be determined despite the larger inaccuracy in the background subtraction.

Direct comparison of the two scattering curves of BSA obtained using the two different IEC–SAXS approaches (Fig. 5 ▸ *d*) shows two very similar curves. Owing to the lower protein concentration at the peak, the curve resulting from the step-gradient method is noisier. However, from about 2.5 nm^−1^ onwards the signal determined using the linear gradient method decreases more steeply. In particular, above 3.5 nm^−1^ the signal determined using the step gradient turns upward. This increase also results in a clear deviation from the predicted signal and is responsible for the higher χ^2^.

### 3.3. IEC–SAXS of the helicase–primase protein D5_323–785_ using a stepwise salt gradient   

To demonstrate the applicability of this approach to a novel sample, we selected D5_323–785_, the D5N and helicase domains of the *Vaccinia virus* helicase–primase D5. The protein fragment forms a hexamer (320.88 kDa) that is required for its activity (Hutin *et al.*, 2016 ▸). After two nickel columns and a size-exclusion chromatography step, a contaminant still persisted and required an additional purification step *via* ion-exchange chromatography (Fig. 2 ▸ *b*), followed by an additional size-exclusion chromatography step. Each step takes time and about 30–50% of the material is lost in total. To optimize the data-collection strategy it would be advantageous to measure the hexamer directly from the Uno Q-1R column.

**Figure 6:**
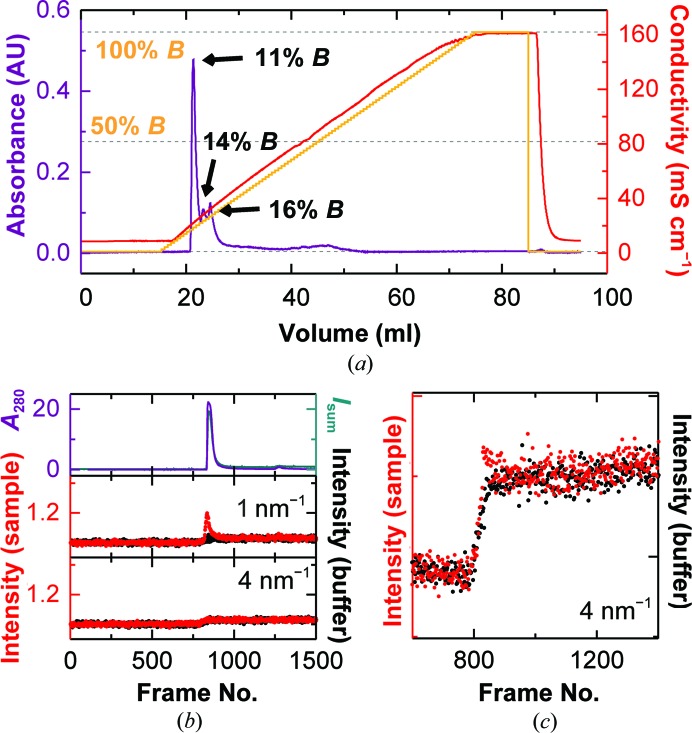
IEC–SAXS of D5_323–785_. (*a*) Ion-exchange chromatogram of D5_323–785_ on the Uno Q-1R column with the three peaks indicated. Orange line, percentage of buffer *B*; red line, the resulting conductivity; violet line, absorbance at 280 nm. (*b*) IEC–SAXS chromatograms of D5_323–785_ using a stepwise salt gradient. Top panel, UV absorbance (violet) and total scattering intensity (green). Middle and bottom panels, chromatograms of sample (red) and buffer (black) runs at *q* = 1 nm^−1^ (middle) and 4 nm^−1^ (bottom). (*c*) Enlargement of the lower panel in (*b*): in the protein run (red) the signal at high *q* overshoots in comparison to the buffer run.

In the standard offline IEC experiment using a linear gradient, three not completely separated peaks at 11, 14 and 16% high-salt buffer were observed (Fig. 6 ▸ *a*).

When using a stepwise gradient a sharp peak is obtained (Fig. 6 ▸ *b*) at 12% salt buffer (142 m*M* NaCl) and small additional peaks in the subsequent steps. This indicates that D5_323–785_ elutes completely at 142 m*M* NaCl and the peaks are better separated than in the linear-gradient elution procedure (Fig. 2 ▸ *a*). The scattering at high *q* (4 nm^−1^) slightly overshoots the scattering level expected from the buffer run (Fig. 6 ▸ *c*), probably owing to the co-elution of small ions. To check that the sample composition does not change throughout the peak, the low-salt buffer from each frame was subtracted and their radii of gyration were compared. This operation is not affected by the small change of background throughout the peak (data not shown). Based on the forward scattering from this preliminary processing, the maximum protein concentration is estimated to be 3.4 mg ml^−1^, corresponding to a 20-fold increase from the injected concentration of 0.17 mg ml^−1^.

Owing to the irregular changes in the background signal that were observed in these data, the approaches to modelling the change in background signal on a frame-by-frame basis that were applied to the previous examples are not applicable. To overcome this issue, first the frames in the peak were averaged in order to improve the signal-to-noise ratio in the high-*q* region, and a matching buffer from a salt-concentration series measured directly before the ion-exchange run was then chosen by comparing the average scattering in the *q*-range between 4.25 and 4.75 nm^−1^.

**Figure 7:**
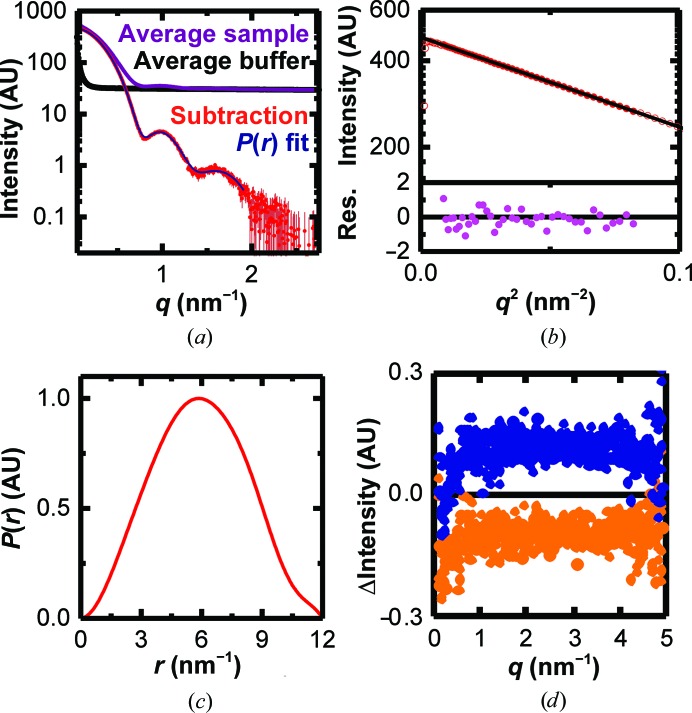
(*a*) Average sample (violet) and buffer (black) curves for the D5_323–785_ protein, the resulting subtraction (red) and the fit for calculating the *P*(*r*) function (blue). (*b*) Guinier fit (black line) of the scattering curve shown in (*a*). Open symbols represent points that were not used for fitting. The lower panel shows the residuals. (*c*) Pair distance distribution function of the average curve, showing a single peak. (*d*) Differences in scattering between the curve based on the best-matching buffer and a clearly under-subtracted curve (preceding buffer step, orange) and over-subtracted curve (subsequent buffer step, blue).

The resulting curve (Fig. 7 ▸ *a*) has a radius of gyration of 4.7 nm (Fig. 7 ▸ *b*) and a Porod volume of 577 ± 5 nm^3^, corresponding to a mass of 338 kDa (Petoukhov *et al.*, 2007 ▸), matching the hexamer mass of 320 kDa well (see also Table 1 ▸). The *P*(*r*) function (Fig. 7 ▸ *c*) shows one peak that is slightly shifted to larger distances. This shape, in addition to the clear minima, hints at a mostly spherical shape with a cavity and matches the expected molecular shape well (Hutin *et al.*, 2016 ▸).

As the steps were chosen more finely than in the previous experiment with BSA, it is easier to estimate the degree of buffer mismatch in the D5_323–785_ experiment. The extent to which it influences the interpretation of the results can be estimated by selecting buffers from a higher and a lower salt step of the buffer run (Fig. 7 ▸ *d*). In both cases the resulting curve is distinguished from the reported curve by a small constant above *q* = 0.8 nm^−1^. Below *q* = 0.8 nm^−1^ the deviations are no longer constant but are below 0.1% of the signal, and therefore do not influence the interpretation.

### 3.4. Additional considerations   

One of the major difficulties in online SEC–SAXS is the effect of radiation damage on the observed signal. The continuously changing signal makes assessment of radiation-induced changes to the SAXS signal difficult. In addition, radiation-damaged material often displays a tendency to adhere to the surfaces of the sample environment (Brookes *et al.*, 2013 ▸; Jeffries *et al.*, 2015 ▸). IEC–SAXS can be performed at relatively high flow rates (1 ml min^−1^ for the studies in this publication); consequently, the average dwelling time of material in the X-ray beam is shorter than for standard SEC–SAXS experiments and the contribution of damaged material to the signal is reduced. Furthermore, the higher flow rate reduces the risk of damaged material spoiling the sample environment by adhering to the surface (Epstein, 1997 ▸).

## 4. Conclusions   

For many macromolecules in solution, ion-exchange chromatography is a required step and is the best-suited purification method, as it can often separate similarly sized proteins. However, the IEC step is usually followed by another purification step, or dialysis, to ensure optimal buffer subtraction. Here, we demonstrate for the first time the application of ion-exchange chromatography directly prior to SAXS measurements.

The linear gradient method presented here is analogous to a standard ion-exchange protocol and does not inherently require additional offline tests. In addition, background correction can be achieved by correct alignment of a buffer run without any need for interpolation. As each frame can be corrected individually, it is possible to confirm a stable signal throughout the peak. However, owing to the continuity of the gradient, sharp and well separated peaks are beneficial.

By carefully selecting the salt concentration of the steps in the step-elution method, this method allows the separation of the peaks to be improved, as no limit exists on the number of substeps. However, background subtraction requires more caution, as the discontinuity of the elution gradient results in the discontinuous co-elution of small ions. In our first example (BSA), it was still possible to correct the background individually for each frame by interpolating the buffer signal. In our second example (D5_323–785_) it was not possible to interpolate the background throughout the peak, and the correction by framewise comparisons was not applicable. Nevertheless, the correct background for the average signal can be found by measuring a variety of mixing ratios prior to the experiment and carefully choosing the matching one. Despite this difficulty, we are confident that it can be applied to a wide variety of biological macromolecules.

Online ion-exchange chromatography adds another important biochemical purification method to the repertoire of purification methods which can be coupled with SAXS (Round *et al.*, 2013 ▸; David & Pérez, 2009 ▸; Graewert *et al.*, 2015 ▸; Jensen *et al.*, 2010 ▸; Hynson *et al.*, 2015 ▸; Mathew *et al.*, 2004 ▸; Watanabe & Inoko, 2009 ▸). Proteins of similar apparent molecular weight, but different net surface charges, can now be separated online using the IEC–SAXS method. While background subtraction is slightly less straightforward than for SEC–SAXS, the variation in the background is smaller than for differential ultracentrifugation coupled with SAXS owing to the high sugar content of the latter (from 15 to 35% sucrose; Hynson *et al.*, 2015 ▸). The background-correction method here for D5_323–785_, based on averages, can be performed using any software package for SAXS data analysis, such as *PRIMUS* (Konarev *et al.*, 2003 ▸) or *SCÅTTER*, assuming that the possible buffer range has been well determined experimentally.

For IEC, buffer conditions, such as salt concentrations or pH, are primarily determined by the biochemical characteristics of the protein of interest, such as its isoelectric point and solubility (Yigzaw *et al.*, 2009 ▸). However, one should aim to minimize the amounts of all additives in order to maximize the X-ray contrast between the protein and the buffer in a SAXS experiment.

As with any SAS data, IEC–SAXS results need to be carefully validated. Special care should be paid to sample monodispersity, which cannot be directly assessed by IEC–SAXS, and correct background subtraction, especially in the case of flexible proteins (Jacques *et al.*, 2012 ▸; Jacques & Trewhella, 2010 ▸). It is important to estimate how mis-subtraction would affect the conclusions drawn from the data. For the results shown in this paper, we have shown that deviations from the ideal background subtraction are small enough to allow reliable conclusions.

In conclusion, IEC–SAXS is a useful technique that allows accurate data collection with fewer preparation steps and minimizing the loss of time and sample. Proof of principle of a simple elution method is demonstrated in this paper with the option to optimize species separation by using steps in the salt gradient. Validation of three different approaches to achieve optimal background subtractions is also given. A suitable combination of elution method and background subtraction can be chosen to suit the sample of interest best and to provide necessary information for data validation (as demonstrated), so that the resulting data can be used with confidence for subsequent analysis and modelling using the standard tools available within the scientific community.

The online SEC system installed at BM29 can routinely perform these experiments and is available for user access on request.

## Supplementary Material

Supporting Information.. DOI: 10.1107/S2059798316012833/wa5110sup1.pdf

## Figures and Tables

**Table 1:** Parameters of SAXS data acquisition and analysis

Data-collection parameters	
Instrument	BM29, ESRF
Wavelength (Å)	0.99
*q*-range (Å^−1^)	0.0032–0.49
Sample-to-detector distance (m)	2.864
Exposure time per frame (s)	1
Concentration range	n.a.
Temperature (K)	293
Detector	PILATUS 1M (Dectris)
Flux (photons s^−1^)	5 × 10^11^
Beam size (µm)	700 × 700
Structural parameters for BSA, linear gradient	
*I*_0_ (from Guinier) (arbitrary units)	0.0778
*R*_g_ (from Guinier) (Å)	27.4 ± 0.1
*q*_min_ *R*_g_ − *q*_max_ *R*_g_ used for Guinier	0.31–1.28
Theoretical *R*_g_ (from *CRYSOL*) (Å)	27.15
Porod volume *V*_p_ (from *SCÅTTER*) (Å^3^)	(116 ± 5) × 10^3^
Molecular mass *M*_r_ (from *V*_p_) (kDa)	68.2
Calculated monomeric *M*_r_ from sequence (kDa)	66.5
Structural parameters for BSA, step gradient	
*I*_0_ (from Guinier) (arbitrary units)	0.0183
*R*_g_ (from Guinier) (Å)	27.1 ± 0.1
*q*_min_ *R*_g_ − *q*_max_ *R*_g_ used for Guinier	0.27–1.29
Theoretical *R*_g_ (from *CRYSOL*) (Å)	27.15
Porod volume *V*_p_ (from *SCÅTTER*) (Å^3^)	(116 ± 5) × 10^3^
Molecular mass *M*_r_ (from *V*_p_) (kDa)	68.2
Calculated monomeric *M*_r_ from sequence (kDa)	66.5
Structural parameters for D5_323–785_, step gradient	
*I*_0_ (from Guinier) (arbitrary units)	0.386
*R*_g_ (from Guinier) (Å)	46.5 ± 0.1
*q*_min_ *R*_g_ − *q*_max_ *R*_g_ used for Guinier	0.44–1.29
*I*_0_ [from *P*(*r*)]	0.378
*R*_g_ [from *P*(*r*)] (Å)	44.7 ± 0.1
*D*_max_ [from *P*(*r*)] (Å)	120
*q*-range used for *P*(*r*) (Å^−1^)	0.03–0.18
Porod volume *V*_p_ (from *SCÅTTER*) (Å^3^)	(577 ± 5) × 10^3^
Molecular mass *M*_r_ (from *V*_p_) (kDa)	338
Calculated monomeric *M*_r_ from sequence (kDa)	321
