# Supplementary material for: Online ion-exchange chromatography for small-angle X-ray scattering
Source: Acta Crystallogr D Struct Biol. 2016 Sep 15;72(Pt 10):1090–9. doi: 10.1107/S2059798316012833 (PMC5053136; doi:10.1107/S2059798316012833)
Supplement: Supplementary file 1 [file d-72-01090-sup1.pdf]

STRUCTURAL
BIOLOGY

Volume 72 (2016)

Supporting information for article:

Online ion-exchange chromatography for small-angle X-ray scattering

Stephanie Hutin, Martha Brennich, Benoit Maillot and Adam Round

S1. SEC-SAXS on BSA

To estimate whether the differences between scattering predicted from the BSA crystal structure and the scattering found by IEC-SAXS are due to systematic errors of the method or due to crystal artefacts, we performed SEC-SAXS on BSA, using a buffer matching the elution point for BSA in the IEC experiment (20 mM Tris pH 7, 142 mM NaCl, 5% glycerol and 1 mM DTT). 500 µL of 10 mg/mL BSA (i.e. 5 mg in total) were injected on a Superdex 200 10/300 GL column (GE Healthcare). The flow rate was 0.5 mL/min. 35 frames with stable signal from the peak corresponding to monomeric BSA were merged to provide the final curve. The resulting curve has a radius of gyration of 2.7± 0.1 nm and a Porod volume of 118 ± 5 nm^3. Comparison to the monomeric crystal structure (pdb entry 3V03)(Majorek *et al.*, 2012) with CRYSOL (Svergun *et al.*, 1995) shows overall agreement with small, but systematic differences in the mid-*q* region (χ^2 = 2.4, figure S1b).

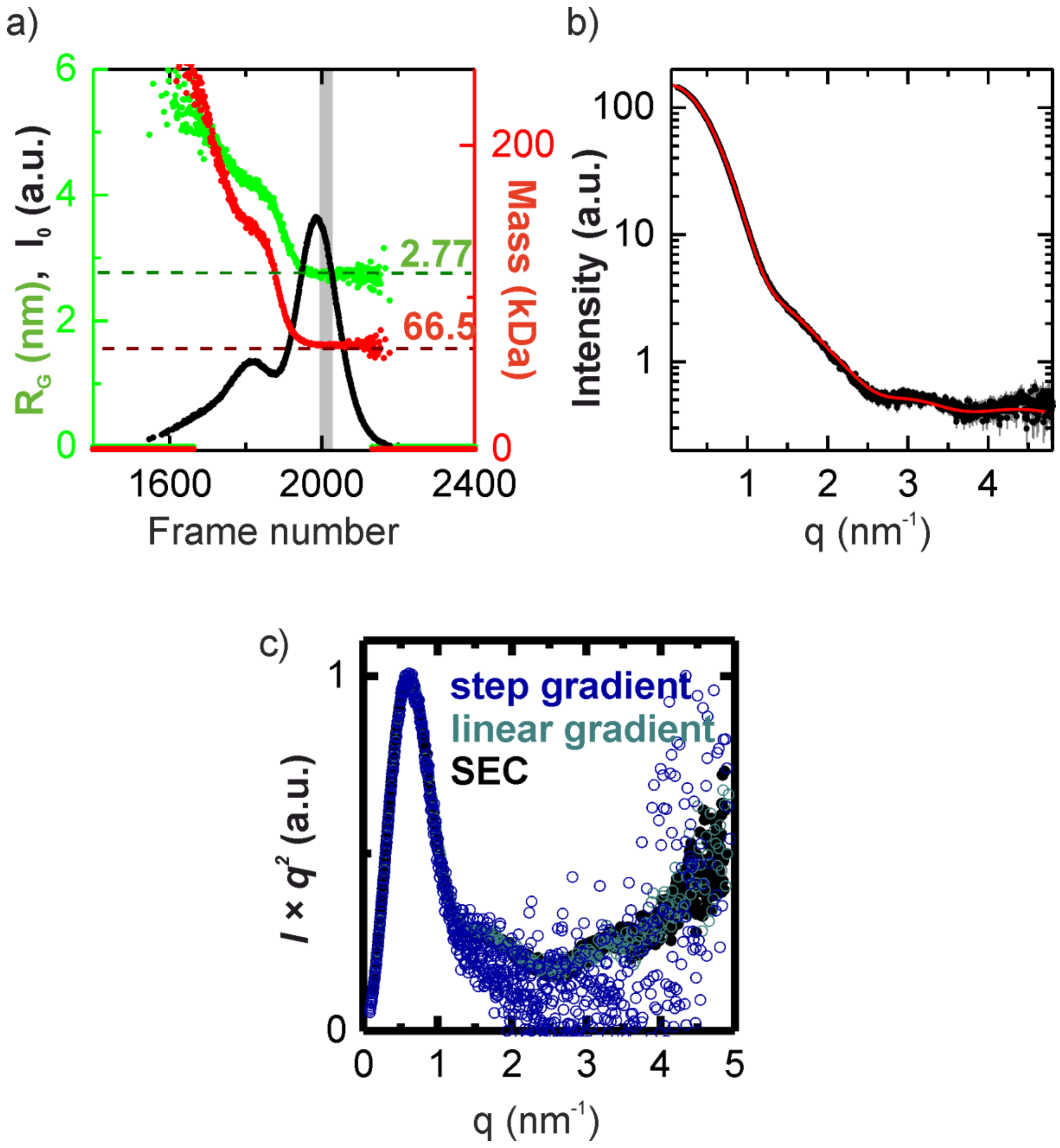

Figure S1 SEC-SAXS of BSA. a) Forward scattering (black), radius of gyration (green) and mass based on the correlated volume (red) for the background corrected curves in the region of interest The grey area indicates which frames were used for subsequent averaging. b) .Fit to the monomeric crystal structure of BSA (pdb entry 3V03 (Majorek *et al.*, 2012)). c) Kratky-plots of BSA collected using SEC-SAXS (black), IEC-SAXS with a linear gradient (green) and IEC-SAXS with a step gradient (blue). The curves were scaled such that the peaks are at the same height.

Data-collection parameters	
Instrument:	ESRF BM29
Wavelength (Å)	0.99
q-range (Å^{-1})	0.0032 – 0.49
Sample-to-detector distance	2.867m
Exposure time (sec)	1 per frame
Concentration range	n.a.
Temperature (K)	293
Detector	Pilatus 1M (Dectris)
Flux (photons/s)	10^{12}
Beam size (μm^2)	700 × 700
Structural parameters for BSA, SEC	
I_0 (cm^{-1}) [from Guinier]	0.12
Rg (Å) [from Guinier]	27.1 ± 0.1
$q_{min}R_g$ – $q_{max}R_g$ used for Guinier	0.36- 0.92
Theoretical Rg (Å) [from Crysol]	27.15
Porod volume V_p (Å^3) [from Scåtter]	(118 ± 5)·10^3
Molecular mass M_r (kDa) [from V_p]	68.2
Calculated monomeric Mr from sequence (kDa)	66.5

Table 1 Parameters of SEC-SAXS data acquisition and analysis

Majorek, K. A., Porebski, P. J., Dayal, A., Zimmerman, M. D., Jablonska, K., Stewart, A. J., Chruszcz, M. & Minor, W. (2012). *Molecular immunology* **52**, 174-182.

Svergun, D., Barberato, C. & Koch, M. (1995). *Journal of applied crystallography* **28**, 768-773.
